# Coronary Computed Tomography Angiography for the Diagnosis and Revascularization Guidance of Coronary Bifurcation Lesions: A Contemporary Review

**DOI:** 10.3390/jcm15124565

**Published:** 2026-06-12

**Authors:** Niya Mileva, Dobrin Vassilev, Panayot Panayotov, Slawomir Golebiewski, Gianluca Rigatelli, Robert J. Gil

**Affiliations:** 1 Medica Cor Hospital, Riga 35 Str, 7013 Ruse, Bulgaria; 2 Faculty of Cardiology, Pulmonology and Endocrinology, Medical University of Pleven, 5800 Pleven, Bulgaria; 3 Faculty of Public Health and Health Care, Ruse University “Angel Kanchev”, 7000 Ruse, Bulgaria; 4 Department of Interventional Cardiology and Internal Diseases, Military National Research Institute, Zegrzynska 8 Str, 05-118 Legionowo, Poland; 5 Cardiology Department, Ospedali Riuniti Padova Sud, 35-121 Padova, Italy; 6 Cardiology Department, National Medical Institute of Internal Affairs and Administration Ministry, 02-507 Warsaw, Poland

**Keywords:** cardiovascular imaging, computed tomography angiography, coronary bifurcations, revascularization planning

## Abstract

**Background**: Coronary bifurcation lesions represent one of the most technically demanding scenarios in coronary artery disease (CAD), associated with higher procedural complexity, restenosis, and periprocedural complications. Recent advances in coronary computed tomography angiography (CCTA) have markedly improved its ability to visualize complex coronary anatomy, assess plaque morphology, and guide revascularization. **Objectives**: This review summarizes (1) technological advances in CCTA over the last decade, (2) its role in evaluating bifurcation stenosis, (3) assessment of plaque morphology and distribution, (4) quantification of bifurcation geometry, and (5) emerging evidence supporting its application in revascularization planning and guidance. **Findings**: Modern wide-detector and dual-source CT systems, iterative and deep-learning reconstruction algorithms, and photon-counting CT (PCCT) have significantly improved temporal and spatial resolution, reduced blooming artifacts, and lowered radiation dose. CCTA now reliably quantifies bifurcation stenosis and plaque distribution, characterizes high-risk plaque features, and accurately measures bifurcation angles. The integration of CT-derived fractional flow reserve (FFR-CT) and artificial intelligence (AI)-based plaque quantification further strengthens its diagnostic and prognostic performance. CCTA-derived bifurcation scores and 3D modelling support procedural strategy selection, stent sizing, and side-branch (SB) protection. **Conclusions**: CCTA has evolved into a comprehensive tool for non-invasive diagnosis, physiological assessment, and pre-procedural planning of bifurcation disease. With the advent of PCCT and AI-enhanced quantitative tools, CCTA is poised to become a central component of revascularization decision-making in complex coronary bifurcations.

## 1. Introduction

Coronary bifurcation lesions represent a distinct and technically demanding subset of coronary artery disease, accounting for approximately 15–20% of all percutaneous coronary interventions (PCI) and continuing to be associated with higher rates of procedural complexity, adverse events, and restenosis [1,2,3]. By definition, these lesions involve the division of a main vessel into one or more side branches (SB), creating a unique anatomical and hemodynamic environment. The interplay of altered shear stress, turbulent flow, and vessel geometry promotes asymmetric plaque deposition, often with preferential involvement of the lateral walls and relative sparing of the carina [4,5]. This heterogeneity, combined with the need to preserve both main vessel and SB patency, makes bifurcation lesions particularly challenging in terms of both accurate diagnosis and optimal revascularization strategy selection [5,6].

Despite its central role in guiding PCI, conventional coronary angiography is inherently limited by its two-dimensional representation of a complex three-dimensional structure [7]. It provides only a luminographic silhouette and lacks the ability to adequately characterize plaque composition, vessel wall remodelling, lesion length, or the true spatial relationship between the main vessel and SB [8]. These limitations are especially pronounced in bifurcation lesions, where angiographic foreshortening, overlap, and suboptimal projection angles can lead to misinterpretation of lesion severity and SB involvement [9]. Consequently, reliance on angiography alone may result in suboptimal procedural planning and stent deployment strategies.

To overcome these limitations, intracoronary imaging modalities such as intravascular ultrasound (IVUS) and optical coherence tomography (OCT) have been increasingly utilized [10]. These techniques provide high-resolution cross-sectional images, enabling detailed assessment of plaque morphology, calcium distribution, vessel dimensions, and stent apposition. However, their invasive nature, requirement for coronary instrumentation, additional procedural time, and cost restrict their use primarily to intra-procedural optimization rather than pre-procedural planning [11]. Moreover, their field of view is limited to the interrogated segment, and they do not provide a comprehensive overview of the entire coronary tree.

Coronary computed tomography angiography (CCTA) has emerged as a powerful non-invasive imaging modality that addresses many of these limitations [12]. By providing high-resolution, three-dimensional visualization of both the coronary lumen and vessel wall, CCTA enables comprehensive assessment of coronary anatomy, plaque burden, and lesion morphology before invasive intervention. Over the past decade, substantial advances in scanner technology, including improved temporal and spatial resolution, iterative reconstruction algorithms, and motion correction techniques, have significantly enhanced image quality while reducing radiation exposure [13]. In parallel, the integration of computational fluid dynamics and machine learning has enabled the derivation of functional indices such as CT-derived fractional flow reserve, allowing simultaneous anatomical and physiological assessment of coronary lesions [14,15].

**Figure 1:**
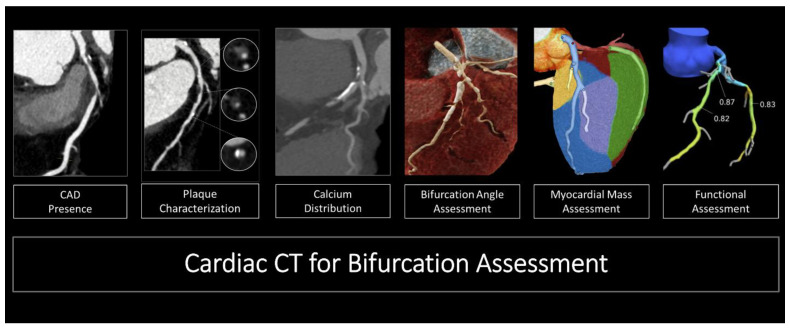
Application of Cardiac Computed Tomography Angiography for coronary bifurcation assessment. CAD—coronary artery disease; CT—computed tomography.

Importantly, CCTA offers unique advantages in the evaluation of bifurcation lesions. First of all, CT coronary angiography plays an important role as a gatekeeper for invasive coronary assessment, as it avoids the use of additional contrast material and radiation exposure, as well as the risks of complications from invasive access [16]. It allows precise characterization of bifurcation geometry (including bifurcation angle, vessel diameters, and lesion length), identification of high-risk plaque features, and assessment of disease distribution in both the main vessel and SB, Figure 1 [17]. These capabilities are particularly valuable for procedural planning, including the selection of stenting strategy (provisional versus two-stent techniques), prediction of SB compromise, and optimization of stent sizing and positioning [18]. Furthermore, emerging applications such as virtual stenting simulations and computational modelling of flow dynamics hold promise for individualized treatment planning in complex bifurcation anatomy [19].

This review aims to provide a comprehensive overview of the evolving role of CCTA in the assessment and management of coronary bifurcation lesions. We focus on recent technological advances, current evidence supporting its diagnostic and prognostic utility, and its potential to guide revascularization strategies, ultimately bridging the gap between non-invasive imaging and interventional decision-making (Table 1).

## 2. Advances in Coronary CT Angiography Technology

### 2.1. Hardware Advancements

Since the introduction of 64-slice CT, modern CCTA has undergone rapid evolution. Wide-detector scanners (256–320 rows) allow single-heartbeat acquisition, reducing misregistration artifacts and improving volumetric accuracy, particularly important in bifurcation regions where motion blurring can distort carinal anatomy [27]. Dual-source CT (DSCT) systems further enhance temporal resolution (<70 ms) and enable high-pitch spiral acquisition at low radiation dose. The most transformative development has been photon-counting CT (PCCT), which directly converts x-ray photons to electronic signals, minimizing electronic noise and enabling true multi-energy imaging. PCCT provides higher spatial resolution (up to 0.25 mm), reduced calcium blooming, and improved plaque tissue differentiation compared with energy-integrating detectors (EID-CT) [14]. Early comparative studies show increased diagnostic confidence for small-caliber vessels and complex bifurcations with PCCT [28,29].

Photon-counting CT (PCCT) represents one of the most important recent technological advances in coronary CT angiography, offering substantial improvements in spatial resolution, signal-to-noise ratio, and plaque characterization compared with conventional energy-integrating detector CT systems. Owing to its ultra-high spatial resolution and reduction of calcium blooming artifacts, PCCT enables more accurate visualization of small-caliber vessels, side-branch ostia, and complex bifurcation anatomy, which are frequently challenging to assess using standard CCTA techniques. These advantages are particularly relevant in coronary bifurcation lesions, where precise delineation of the carina, plaque extension into the side branch, and vessel geometry are essential for procedural planning. Recent studies have demonstrated that PCCT improves diagnostic confidence in heavily calcified lesions and enhances assessment of non-calcified and low-attenuation plaque components, allowing more reliable evaluation of plaque vulnerability and distribution within bifurcation segments.

Beyond anatomical visualization, plaque localization and composition derived from advanced CCTA and PCCT imaging may have direct implications for revascularization strategy selection. Plaque burden involving the side-branch ostium or plaque located proximal to the carina has been associated with an increased risk of side-branch compromise during main vessel stenting. Similarly, extensive lipid-rich or high-risk plaque distribution may predict plaque shift, distal embolization, or no-reflow phenomena during PCI. Detailed preprocedural assessment of these features may therefore support individualized strategy selection, including the choice between provisional stenting and planned two-stent techniques. In addition, improved characterization of plaque extent and vessel wall morphology may facilitate more accurate determination of stent landing zones, minimizing geographic miss and reducing the risk of edge dissection or incomplete lesion coverage. Consequently, PCCT-enhanced plaque assessment has the potential to further strengthen the role of CCTA as a comprehensive tool for bifurcation PCI planning and procedural risk stratification.

### 2.2. Image Reconstruction and Motion Correction

Iterative reconstruction techniques (adaptive statistical iterative reconstruction, model-based iterative reconstruction) and deep-learning image reconstruction (DLIR) have markedly reduced image noise, enabling diagnostic quality at radiation doses below 1 mSv [30]. Vendor-specific motion-correction algorithms correct for motion across cardiac phases, particularly improving the distal and bifurcation segments that were previously prone to artifacts.

### 2.3. Dose Efficiency and Contrast Optimization

Automatic exposure control, individualized tube potential (kVp) selection, and low-concentration contrast protocols have further reduced patient radiation and iodine dose without compromising quality [31,32]. When combined with high-pitch acquisition, total scan times are <0.3 s—effectively freezing coronary motion even at higher heart rates. These technical improvements translate directly into better delineation of side branch ostia, carina structure, and plaque eccentricity, which are critical for bifurcation analysis [17].

## 3. Assessment of Coronary Bifurcation Stenosis

### 3.1. Accuracy and Correlation with Invasive Angiography

Several studies have demonstrated the high diagnostic accuracy of CCTA for detecting significant stenosis in bifurcation lesions compared with invasive coronary angiography (ICA) and fractional flow reserve (FFR) [19,27,28]. CCTA offers the advantage of assessing both luminal narrowing and wall morphology, which allows differentiation between true anatomical narrowing and apparent narrowing due to remodelling. Medina classification, traditionally based on angiography, can be reliably derived from CCTA [29]. Grodecki et al. [20] reported good concordance between CCTA and ICA in Medina classification, and true bifurcation lesions classified as involving both the main vessel (MV) and SB (Medina 1, 1, 1; 1, 0, 1; 0, 1, 1) were predictive of SB occlusion after PCI. CCTA can also assess lesion length and plaque distribution along both branches, parameters not well captured on 2D angiography [31].

### 3.2. Functional Assessment with Computational Fluid Dynamics-Based Technologies

CCTA can be coupled with computational fluid dynamics to estimate CT-derived fractional flow reserve (FFR-CT). Large-scale multicentre trials such as NXT and PLATFORM demonstrated high diagnostic performance of FFR-CT compared with invasive FFR [33,34]. Subgroup analyses have shown that FFR-CT maintains reliability even in bifurcation lesions, where local flow separation may create complex hemodynamic. The ability of FFR-CT to provide lesion-specific physiology enhances bifurcation evaluation beyond anatomic stenosis alone. Studies have shown that a significant FFR-CT drop across the MV–SB junction correlates with adverse outcomes and can guide the necessity of intervention [35].

Beyond FFR-CT, emerging CT-based computational physiology platforms incorporating quantitative flow ratio (QFR)-like principles and virtual flow modelling have further expanded the functional capabilities of coronary CT angiography. These software solutions integrate three-dimensional coronary anatomy derived from CCTA with computational fluid dynamics, contrast-flow modelling, or reduced-order flow simulations to estimate lesion-specific pressure gradients and coronary flow impairment noninvasively. Similarly to angiography-derived QFR, these CT-based approaches aim to provide rapid functional assessment without the need for invasive pressure-wire interrogation or pharmacologic hyperemia. In bifurcation lesions, where vessel geometry, side-branch flow distribution, and plaque localization substantially influence coronary hemodynamics, CT-based QFR platforms may offer important additional information regarding the physiological significance of both the main vessel and side branch. Preliminary studies suggest that these technologies can improve identification of functionally relevant stenoses, predict side-branch compromise after main vessel stenting, and support individualized procedural planning. Furthermore, integration of CT-derived anatomical modelling with virtual PCI simulation may allow prediction of post-interventional flow dynamics and optimization of bifurcation treatment strategies before invasive intervention. Although these techniques remain under active clinical investigation and require further validation in complex bifurcation anatomy, they represent a promising step toward fully integrated non-invasive anatomical and physiological planning of coronary bifurcation PCI.

## 4. Plaque Morphology and Distribution in Coronary Bifurcations

### 4.1. High-Risk Plaque Features

Coronary computed tomography angiography (CCTA) enables comprehensive, non-invasive characterization of coronary atherosclerotic plaque, with particular utility in identifying high-risk plaque (HRP) features associated with future acute coronary syndromes (ACS) [36,37]. Key HRP characteristics detectable by CCTA include low-attenuation plaque (typically defined as <30 Hounsfield units), positive remodelling, spotty calcifications, and the napkin-ring sign, all of which have been linked to histopathological features of vulnerable plaque such as large lipid cores, thin fibrous caps, and increased inflammatory activity [36,38,39].

Among these, low-attenuation plaque has been shown to correlate strongly with necrotic core burden as assessed by intravascular ultrasound (IVUS) and histology [39]. Positive remodelling, defined as an increase in vessel diameter at the lesion site relative to a reference segment, reflects outward compensatory enlargement and is frequently associated with plaque vulnerability and rapid progression. Spotty calcifications, typically small (<3 mm) and scattered deposits, are thought to represent active calcification processes and have been associated with plaque instability. (Table 2) The napkin-ring sign—characterized by a low-attenuation core surrounded by a higher-attenuation rim—is considered one of the most specific CCTA markers of vulnerable plaque and has demonstrated strong associations with thin-cap fibroatheroma on optical coherence tomography (OCT) and histopathology [39].

Large prospective studies have validated the prognostic significance of these HRP features. In the SCOT-HEART trial, the presence of adverse plaque characteristics on CCTA was independently associated with a markedly increased risk of future myocardial infarction, even after adjustment for stenosis severity [40]. Similarly, the PROMISE study demonstrated that incorporation of plaque features improved risk stratification beyond traditional clinical and anatomical assessment [5]. These findings underscore the concept that plaque composition, rather than luminal narrowing alone, is a critical determinant of clinical events [41].

Quantitative plaque analysis represents an important advancement in CCTA, enabling objective measurement of total plaque burden as well as its individual components, including calcified, non-calcified, and low-attenuation plaque volumes [42]. Semiautomated and fully automated software platforms allow reproducible volumetric assessment across the coronary tree, facilitating both cross-sectional and longitudinal evaluation of disease. Multiple validation studies have demonstrated strong correlations between CCTA-derived plaque quantification and intravascular imaging modalities such as IVUS and OCT, particularly for non-calcified and low-attenuation components [43]. These quantitative metrics have also been shown to predict adverse cardiovascular outcomes, with low-attenuation plaque volume emerging as one of the most powerful independent predictors of future events [44].

**Figure 2:**
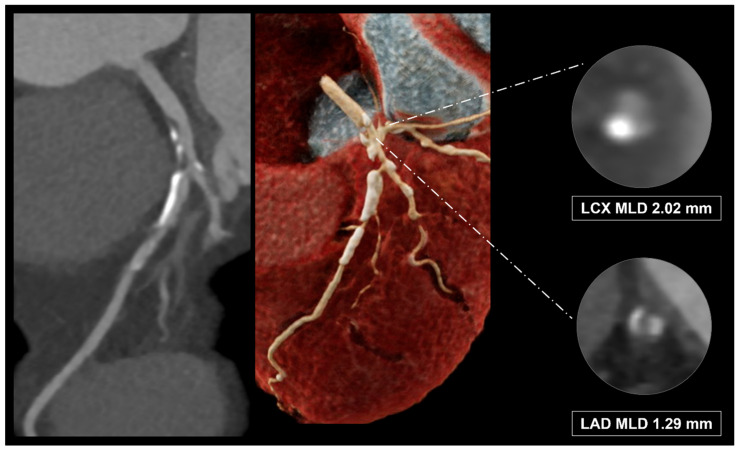
CCTA example demonstrating multiplanar reconstruction and 3D visualization of left coronary artery bifurcation anatomy and plaque distribution. LCX—left circumflex artery; LAD—left anterior descending artery; MLD—minimal lumen diameter.

In the context of coronary bifurcation lesions, HRP assessment by CCTA provides additional insights into the spatial distribution of vulnerable plaque, which is often heterogeneous due to complex flow dynamics [45]. Studies have demonstrated preferential accumulation of high-risk features along the lateral walls of bifurcations, where low wall shear stress promotes atherogenesis and plaque progression. Identification of such features preprocedurally may have important implications for PCI planning, including selection of landing zones and anticipation of procedural complications such as distal embolization or side branch compromise. Overall, CCTA-derived identification and quantification of HRP features provide a powerful tool for integrating anatomical, compositional, and prognostic information, moving beyond traditional stenosis-based assessment toward a more comprehensive evaluation of coronary artery disease, Figure 2.

### 4.2. Plaque Localization and Distribution in the Coronary Bifurcation

In coronary bifurcation anatomy, atherosclerotic plaque distribution is characteristically heterogeneous and asymmetric, reflecting the complex interplay between vascular geometry and local hemodynamic forces. CCTA studies, supported by computational fluid dynamics (CFD) analyses, have consistently demonstrated preferential accumulation of non-calcified and lipid-rich plaque along the lateral walls and outer curvatures of the bifurcation—regions exposed to low and oscillatory endothelial shear stress (ESS) [46]. In contrast, the carina region is typically spared due to exposure to higher, laminar shear stress, which exerts a protective effect against atherogenesis [47]. This spatial pattern of plaque deposition has been confirmed in both imaging and histopathological studies and represents a fundamental feature of bifurcation disease biology [48,49].

Beyond general distribution patterns, the localization of plaque relative to the side branch (SB) ostium has important clinical implications. Plaque burden located proximal to the carina and extending toward or into the SB ostium has been strongly associated with an increased risk of SB compromise during PCI, particularly in provisional stenting strategies [50]. Mechanistically, this may occur through plaque or carina shift during main vessel stent deployment, leading to SB ostial narrowing or occlusion [51]. Both invasive imaging and angiographic studies have shown that larger plaque burden at the SB ostium, greater plaque eccentricity, and the presence of lipid-rich or necrotic-core components are predictors of SB flow deterioration and periprocedural myocardial injury [52].

CCTA offers a unique advantage in this context by enabling comprehensive, non-invasive visualization of plaque distribution across both the main vessel and SB prior to intervention [53]. Through multiplanar and cross-sectional reconstructions, CCTA can delineate plaque extent, eccentricity, and composition, facilitating identification of high-risk anatomical configurations. Quantitative plaque mapping further enhances this assessment by allowing objective measurement of plaque burden in predefined segments, including the proximal main vessel and SB ostium, which are critical regions for procedural planning [53].

The ability of CCTA to accurately characterize plaque distribution in bifurcation lesions has been validated in comparative studies with intravascular imaging. In a study by Radunović A et al. [17], CCTA was directly compared with intravascular ultrasound (IVUS) in patients with true bifurcation lesions. The authors demonstrated good agreement between modalities for both plaque composition and spatial distribution, including identification of eccentric plaques and necrotic-core-rich regions. Importantly, CCTA reliably identified plaque localization relative to the SB ostium and carina, supporting its role in preprocedural planning. Similar findings have been reported in other validation studies, which have shown that CCTA can accurately detect non-calcified plaque and assess plaque eccentricity when compared with IVUS and optical coherence tomography [43,54].

From a clinical standpoint, detailed knowledge of plaque localization may influence PCI strategy selection. For example, extensive plaque burden involving the SB ostium may favour a planned two-stent technique, whereas more limited or distal plaque distribution may support a provisional approach. Additionally, identification of lipid-rich or high-risk plaque near the SB origin may prompt more cautious procedural strategies to minimize distal embolization and no-reflow phenomena. Overall, CCTA-derived assessment of plaque localization and distribution provides critical insights into the pathophysiology of bifurcation lesions and offers valuable information for individualized procedural planning. By integrating geometric and compositional data, CCTA has the potential to improve prediction of SB compromise and optimize revascularization strategies in this complex lesion subset.

### 4.3. Quantitative Plaque Analysis

Modern semi-automated software enables volumetric plaque quantification, color-coded mapping, and tissue-specific thresholds [55]. When applied to bifurcations, these tools allow calculation of plaque volume ratio between MV and SB, aiding the prediction of SB occlusion risk. Serial CCTA studies can also monitor plaque regression or progression following medical therapy, offering a unique opportunity for non-invasive follow-up [56].

## 5. Bifurcation Angle and Geometric Analysis

### 5.1. Measurement Techniques

Coronary computed tomography angiography (CCTA) enables precise three-dimensional (3D) quantification of bifurcation geometry through centerline-based reconstruction of the coronary tree. Using semi-automated or fully automated segmentation algorithms, CCTA allows reproducible measurement of key geometric parameters, including the main vessel–side branch (MV–SB) angle, proximal–distal main vessel angle, bifurcation curvature, and vessel diameter ratios [57]. Compared with conventional coronary angiography, which is limited by two-dimensional projection, vessel overlap, and foreshortening, CCTA provides a true 3D representation of bifurcation anatomy, resulting in significantly improved measurement accuracy and interobserver reproducibility [58].

Several studies have validated CCTA-derived bifurcation angle measurements against invasive imaging and 3D quantitative coronary angiography (3D-QCA) [59]. These investigations have demonstrated strong agreement, with CCTA offering the additional advantage of assessing the entire coronary tree noninvasively [60]. Furthermore, dedicated post-processing workstations enable standardized centreline extraction and automated angle calculation, reducing operator dependency. More recently, artificial intelligence (AI)-based algorithms and deep learning-driven segmentation tools have been introduced, allowing rapid and highly reproducible extraction of coronary geometry, including complex bifurcation parameters [61]. These approaches facilitate large-scale quantitative analyses and may support integration of geometric data into predictive models for procedural planning and risk stratification. Beyond static measurements, CCTA datasets can also be used for advanced computational modelling, including reconstruction of patient-specific coronary anatomy for computational fluid dynamics (CFD) simulations. This capability enables comprehensive evaluation of the interplay between geometry and hemodynamic, which is particularly relevant in bifurcation lesions where small geometric variations may significantly impact flow distribution and lesion progression.

### 5.2. Hemodynamic Implications

Bifurcation geometry plays a critical role in determining local hemodynamic conditions and atherosclerotic plaque distribution. Experimental, computational, and clinical studies have consistently demonstrated that wider bifurcation angles—typically greater than 70–80°—are associated with disturbed flow patterns, including flow separation, recirculation zones, and regions of low and oscillatory wall shear stress (WSS) [62]. These adverse hemodynamic conditions promote endothelial dysfunction, inflammation, and lipid accumulation, leading to preferential plaque formation along the lateral walls of the bifurcation, while the carina is relatively spared due to preserved laminar flow and higher shear stress.

CCTA-based studies incorporating CFD analysis have confirmed these observations in vivo, demonstrating a strong relationship between bifurcation angle and local WSS distribution [6]. In particular, larger bifurcation angles have been linked to greater areas of low WSS and an increased plaque burden. They have also been associated with features of plaque vulnerability, such as positive remodelling and low-attenuation plaque. These findings provide mechanistic insight into the propensity for lesion development and progression in specific regions of the bifurcation.

Importantly, bifurcation geometry also influences the physiological significance of coronary stenoses. Studies integrating CCTA with computed FFR (FFR-CT) have shown that larger bifurcation angles are associated with greater trans-lesional pressure gradients, even in lesions of intermediate angiographic severity [22]. This effect is thought to result from increased flow energy loss due to flow separation and inefficient streamlining at wider angles. Computational models and clinical imaging studies have further demonstrated that the interaction between bifurcation angle, lesion location, and vessel size mismatch can significantly modulate FFR values, underscoring the importance of considering geometric factors when interpreting physiological assessments in bifurcation disease.

From a clinical perspective, these geometric and hemodynamic insights have important implications for PCI planning and outcomes [63]. Wider bifurcation angles have been associated with increased risk of side branch compromise, more complex stenting strategies, and higher rates of restenosis following intervention. Consequently, preprocedural assessment of bifurcation geometry using CCTA may help identify high-risk anatomical subsets and guide the selection of optimal revascularization strategies, including the choice between provisional and two-stent techniques [18]. Overall, CCTA-based geometric analysis provides a robust and noninvasive framework for understanding the structural and functional determinants of coronary bifurcation disease, bridging anatomical characterization with hemodynamic and clinical relevance. These findings suggest that bifurcation angle is not merely a geometric descriptor but a surrogate marker of hemodynamic stress and potential ischemia.

## 6. CCTA for Revascularization Planning and Guidance

### 6.1. Pre-Procedural Strategy Selection

CCTA offers a detailed roadmap for procedural planning. 3D reconstructions display the course, take-off, and diameter of the MV and SB, helping determine whether a provisional or two-stent approach is most appropriate [21,64]. The extent of SB disease and its ostial involvement can be directly visualized. CT bifurcation score—incorporating factors such as plaque presence at the SB ostium, degree of calcification, necrotic-core volume, and MV/SB area ratio—predicts the risk of SB occlusion during MV stenting [65]. Lee et al. demonstrated that a high CT bifurcation score correlated with periprocedural SB loss and worse outcomes, outperforming traditional Medina classification [65].

### 6.2. Stent Sizing and Landing Zone Determination

Accurate lumen and vessel wall measurements derived from CCTA play a central role in guiding stent sizing and the selection of optimal landing zones, with the potential to reduce complications such as edge dissection and geographic miss. Contemporary evidence supports the reliability of CCTA for these purposes. In comparative studies against intravascular imaging, CCTA-derived measurements of distal reference vessel diameter have demonstrated strong agreement with optical coherence tomography (OCT), with substantial concordance between CT-based and OCT-guided stent diameter selection [66]. These findings suggest that non-invasive preprocedural planning using CCTA can approximate the precision traditionally achieved with intracoronary imaging.

Beyond simple diameter assessment, CCTA enables comprehensive evaluation of lesion length, plaque distribution, and vessel remodelling, all of which are critical for accurate landing zone determination. Multiplanar reconstructions and cross-sectional imaging allow identification of relatively disease-free reference segments, facilitating appropriate stent coverage and minimizing the risk of incomplete lesion treatment. This is particularly relevant given prior intravascular imaging studies demonstrating that angiographically “normal” reference segments frequently harbour significant atherosclerotic burden, contributing to edge-related complications and restenosis [67]. By visualizing both lumen and vessel wall, CCTA may improve the detection of subclinical plaque extension and thereby refine landing zone selection.

Several observational and mechanistic studies further highlight the importance of precise landing zone identification in preventing adverse outcomes [68,69]. Data from intravascular imaging registries have shown that edge dissections are associated with residual plaque burden, stent oversizing, and suboptimal reference segment selection [70]. Similarly, incomplete lesion coverage or “geographic miss” has been linked to increased restenosis and target lesion failure, particularly in complex anatomical settings such as ostial and bifurcation lesions. In this context, CCTA has been proposed as a valuable tool for preprocedural identification of optimal stent positioning [71]. For example, studies using high-resolution CCTA have demonstrated its ability to define precise landing zones and detect geographic miss when compared with conventional angiographic assessment, suggesting that angiography alone may underestimate lesion extent.

Importantly, emerging data from CT-guided PCI workflows indicate that CCTA-derived anatomical parameters—including minimal lumen diameter, reference vessel size, and plaque characteristics—can be systematically integrated into structured planning approaches to guide stent sizing and landing zone selection [72,73]. These approaches have shown promise in improving procedural efficiency and reducing uncertainty during intervention, with early studies suggesting potential reductions in contrast use, radiation exposure, and procedural time. In complex lesion subsets, including bifurcations, CCTA-based planning also enables more accurate delineation of plaque distribution across the main vessel and side branch, supporting tailored stenting strategies and minimizing the risk of side branch compromise.

In addition to preprocedural planning, fusion imaging techniques that integrate CCTA-derived three-dimensional coronary reconstructions with live fluoroscopy have been developed to guide real-time stent deployment [74]. Systems such as CT–fluoroscopy co-registration platforms allow dynamic visualization of vessel geometry, lesion extent, and predefined landing zones during PCI, potentially reducing vessel foreshortening and improving spatial accuracy. Overall, accumulating evidence suggests that CCTA provides accurate and reproducible measurements for stent sizing and enables more informed selection of landing zones compared with angiography alone. By incorporating plaque characterization and three-dimensional vessel assessment, CCTA-based planning may reduce edge-related complications and improve procedural outcomes, particularly in anatomically complex lesions such as coronary bifurcations.

### 6.3. Physiologic Planning Using FFR-CT

The integration of computed tomography-derived fractional flow reserve (FFR-CT) into CCTA analysis has further enhanced the functional assessment of coronary bifurcation lesions, addressing a key limitation of purely anatomical imaging. It is well established from invasive physiology studies that angiographic severity correlates poorly with functional significance in bifurcation disease. In a dedicated analysis of bifurcation lesions, only approximately 46% of angiographically significant stenoses were functionally ischemic (FFR ≤ 0.80), underscoring the importance of physiological lesion assessment in this setting [75]. Moreover, the complex interplay between main vessel and side branch disease, myocardial territory, and lesion length influences pressure gradients, making physiological evaluation particularly relevant in bifurcations [76,77].

FFR-CT extends these principles into the non-invasive domain by combining anatomical and computational fluid dynamics-based functional assessment. Large multicentre validation trials such as DISCOVER-FLOW have demonstrated that FFR-CT improves diagnostic accuracy and specificity compared with CCTA alone, with strong correlation to invasive FFR measurements [78]. Although these studies were not specific to bifurcation lesions, they established the foundation for applying FFR-CT in complex coronary anatomies.

More recent investigations have specifically explored the interaction between coronary bifurcation geometry and FFR-CT values. Computational and imaging-based studies have shown that bifurcation angle, vessel caliber mismatch, and flow division significantly influence pressure gradients and FFR-CT measurements. For example, FFR-CT values have been demonstrated to decline as a function of bifurcation angle, even in the absence of significant atherosclerotic disease, suggesting that geometric factors must be considered when interpreting physiological significance in bifurcation segments [63]. These findings highlight the unique hemodynamic environment of bifurcations and the potential for FFR-CT to capture these complex flow patterns noninvasively.

Emerging data also suggest that FFR-CT may improve lesion-specific decision-making in bifurcation PCI planning [19,79]. By providing vessel-specific pressure maps along both the main vessel and side branch, FFR-CT enables identification of functionally significant segments, potentially guiding the need for side branch intervention and supporting a physiology-guided provisional stenting strategy. This approach is conceptually aligned with invasive FFR-guided bifurcation PCI, which has been shown to safely defer unnecessary side branch treatment and reduce procedural complexity without compromising outcomes [19].

In addition, FFR-CT offers the ability to simulate post-intervention physiology and evaluate virtual treatment scenarios [80], including the impact of stenting on both main vessel and side branch flow. Although still largely investigational, such “virtual PCI” applications may be particularly valuable in bifurcation lesions, where treatment strategy selection (e.g., provisional versus two-stent techniques) has important hemodynamic implications. Early computational studies integrating CCTA-derived anatomy and flow modelling suggest that patient-specific simulations can predict changes in coronary physiology following intervention, opening new avenues for individualized procedural planning. Despite these promising developments, several limitations remain. The accuracy of FFR-CT in heavily calcified vessels, small side branches, and complex bifurcation anatomy may be reduced, and standardized thresholds for side branch ischemia are not well established [81]. Furthermore, most clinical validation studies have focused on vessel-level rather than lesion-level or bifurcation-specific outcomes, and prospective trials evaluating FFR-CT-guided strategies in bifurcation PCI are still lacking.

Overall, current evidence suggests that FFR-CT provides a valuable, non-invasive tool for integrating anatomical and functional assessment in coronary bifurcation lesions. By capturing the complex hemodynamic effects of bifurcation geometry and lesion distribution, FFR-CT has the potential to refine risk stratification and guide revascularization strategies beyond what is achievable with anatomy alone.

## 7. Limitations of CCTA in Coronary Bifurcation Lesions

Despite major technological advances, several important limitations continue to affect the application of coronary computed tomography angiography (CCTA) in the assessment of bifurcation lesions. One of the principal challenges remains calcium blooming artifact, particularly in heavily calcified bifurcations and ostial side-branch disease [82]. Dense calcifications may lead to overestimation of stenosis severity, obscuration of lumen boundaries, and reduced accuracy of plaque characterization, especially in small-caliber vessels. Although photon-counting CT (PCCT) and advanced reconstruction algorithms have reduced blooming effects, complete elimination of calcium-related artifacts has not yet been achieved.

Assessment of previously stented bifurcation segments also remains challenging. Metallic stent struts, beam-hardening artifacts, and limited spatial resolution may impair accurate evaluation of in-stent restenosis, stent expansion, and side-branch ostial patency after bifurcation PCI. These limitations are particularly relevant in complex two-stent techniques where overlapping metallic layers may further degrade image interpretability.

Another important limitation concerns evaluation of small side branches (SBs), which often approach the spatial resolution limits of current CT technology. Reduced vessel diameter, motion artifacts, and partial-volume effects may compromise accurate assessment of SB stenosis severity, plaque composition, and physiological significance. Consequently, the diagnostic performance of both CCTA and CT-derived physiological indices may be lower in distal or small-caliber side branches compared with larger epicardial vessels.

Image quality also remains dependent on heart-rate control and rhythm stability [81]. Elevated heart rates, arrhythmias, and respiratory motion may reduce image quality despite advances in motion-correction algorithms and high-temporal-resolution scanners. Although modern dual-source CT and high-pitch acquisition techniques have significantly improved temporal resolution, image degradation may still occur in patients with atrial fibrillation, frequent ectopy, or inability to maintain breath-hold instructions.

CCTA additionally requires exposure to ionizing radiation and iodinated contrast administration. While contemporary dose-reduction techniques have markedly lowered radiation exposure, cumulative radiation burden remains relevant, particularly in younger patients or those requiring serial imaging. Similarly, iodinated contrast use may limit applicability in patients with advanced renal dysfunction or severe contrast allergy.

Quantitative plaque analysis and AI-based plaque characterization introduce further methodological challenges. Plaque quantification may vary according to scanner type, reconstruction technique, segmentation algorithms, and vendor-specific attenuation thresholds used to define calcified, non-calcified, and low-attenuation plaque components. This variability may affect reproducibility and inter-platform comparability, particularly in multicentre studies and longitudinal plaque assessment. Standardization efforts are ongoing but have not yet been universally implemented.

Finally, despite growing evidence supporting FFR-CT and AI-enhanced plaque analysis, the real-world availability of these advanced computational tools remains limited. Many platforms require off-site processing, specialized software infrastructure, additional costs, and expert interpretation, potentially restricting widespread implementation in routine clinical practice. Furthermore, dedicated prospective outcome-based studies specifically evaluating CCTA-guided bifurcation PCI strategies remain limited, and current evidence is still largely extrapolated from broader coronary artery disease populations. Therefore, while CCTA provides substantial anatomical and functional information for bifurcation assessment, its findings should currently be interpreted as complementary to invasive physiology and intracoronary imaging rather than as a complete replacement for these modalities in complex bifurcation intervention planning.

## 8. Conclusions

In the last decade, CCTA has evolved from a purely anatomical imaging tool into a comprehensive diagnostic and interventional planning modality for coronary bifurcation disease. Advances in detector technology, reconstruction algorithms, and computational physiology now allow precise assessment of stenosis severity, plaque composition, bifurcation geometry, and functional significance. When integrated with FFR-CT and AI-driven quantitative analysis, CCTA can predict procedural complexity, inform stenting strategies, and potentially improve clinical outcomes. While CCTA is highly promising for precision procedural planning and integrated anatomical–functional assessment, robust outcome-based validation of CCTA-guided bifurcation PCI strategies remains limited and requires further prospective investigation. Future research should focus on outcome-based validation of CCTA-guided bifurcation revascularization, ensuring this technology becomes a cornerstone of precision coronary intervention.

## Figures and Tables

**Table 1:** Key Recent Studies on CCTA in Bifurcation Lesions.

Study (Year)	Design/n	Main Focus	Key Findings
Grodecki et al., 2020 [20]	102 bifurcation lesions	CCTA vs. ICA Medina classification	Good concordance; Medina (1, 1, 1) predictive of SB occlusion
Lee et al., 2018 [21]	115 patients	CT bifurcation score	Higher score predicted SB occlusion during PCI
Radunović et al., 2023 [17]	80 lesions	CCTA vs. IVUS	Strong agreement for plaque composition and distribution
Tsugu et al., 2022 [22]	156 patients	Bifurcation angle vs. FFR-CT	Larger angle associated with lower FFR-CT
Si-Mohamed et al., 2022 [14]	Comparative imaging	PCCT vs. EID-CT	Higher resolution and confidence, less blooming
Wolny et al., 2017 [23]	Randomized study, 92 patients	PCI planned based on coronary angiography alone vs. CCTA and angiography	CTA-assisted bifurcation PCI lead to similar immediate results, however, is associated with higher use of single-stent procedures and less SB stenting.
Dawson et al., 2022 [24]	Review	HRP features on CCTA	Standardization of HRP metrics for risk stratification
Sandoval et al., 2025 [25]	Clinical workflow	CCTA-guided PCI planning	Improved strategy selection and procedure efficiency
Carvalho et al., 2025 [19]	Case series, 3 patients	CTA-guided bifurcation PCI with the FFR_CT_ planner	Preprocedural planning with coronary CTA and FFR_CT_-based applications including virtual PCI and myocardial mass can facilitate and optimize bifurcation planning.
Opolski et al., 2020 [26]	Prospective study, 363 patients with 400 bifurcation lesions	CTA-derived RESOLVE score for predicting SB occlusion in coronary bifurcation intervention	CTA-derived RESOLVE score was accurate and reliable for prediction of SB occlusion in coronary bifurcation intervention.

**Table 2:** Main coronary bifurcation lesion parameters assessable by CCTA.

Domain	Parameter	Description/Definition	Clinical Relevance for PCI Planning
Anatomical geometry	Main vessel (MV) diameter	Reference lumen/vessel diameter proximal and distal to bifurcation	Guides stent sizing and selection
	Side branch (SB) diameter	Diameter of SB at ostium and reference segment	Determines clinical significance and need for SB protection
	Lesion length (MV/SB)	Longitudinal extent of atherosclerotic plaque	Determines stent length and landing zones
	Bifurcation angle (MV–SB)	Angle between MV and SB centrelines	Predicts flow disturbance, SB compromise risk, and stenting strategy
	Proximal–distal MV angle	Curvature of the main vessel across bifurcation	Influences stent conformability and expansion
	Vessel tapering	Change in vessel diameter from proximal to distal MV	Important for appropriate stent sizing (e.g., tapered stents)
	Carina position and morphology	Geometry of flow divider between MV and SB	Influences risk of carina shift during PCI
Plaque burden and composition	Total plaque volume	Overall atherosclerotic burden within bifurcation segment	Predictor of procedural complexity and outcomes
	Non-calcified plaque volume	Lipid-rich/fibrous plaque component	Associated with plaque vulnerability and embolization risk
	Low-attenuation plaque (<30 HU)	Surrogate for necrotic core	Marker of high-risk plaque (HRP)
	Calcified plaque burden	Extent and distribution of calcium	Predicts stent underexpansion and need for lesion preparation
	Spotty calcifications	Small focal calcium deposits	Associated with plaque instability
	Plaque eccentricity	Asymmetric plaque distribution within vessel wall	Predicts SB compromise and stent expansion issues
Plaque localization	Plaque at SB ostium	Presence and extent of plaque at SB origin	Strong predictor of SB occlusion during PCI
	Plaque proximal to carina	Plaque upstream of bifurcation	Associated with plaque shift after stenting
	Lateral wall vs. carina involvement	Spatial distribution of plaque	Reflects shear stress patterns and procedural risk
High-risk plaque features	Positive remodelling	Ratio of the vessel’s diameter (or area) at the site of the plaque to the diameter of a normal, reference section (remodelling index, (RI))—greater than 1.1.	Marker of vulnerable plaque
	Napkin-ring sign	Low-attenuation core with higher attenuation rim	Highly specific for high-risk plaque
	Spotty calcification	-Less than 3 mm at its largest dimension. -The calcium arc occupies less than 90 degrees of the vessel’s circumference.	Marker of vulnerable plaque
Bifurcation angle assessment	Angle of SB take off		Identifies predictors of SB occlusion after MV stenting; Steep SB take-off angles can predict difficulty in SB wiring or balloon delivery during PCI.
Functional assessment	FFR-CT (MV and SB)	Non-invasive pressure-derived ischemia assessment	Identifies functionally significant lesions and guides revascularization
	Pressure drop across bifurcation	Trans lesional gradient along MV/SB	Helps determine need for SB intervention
Hemodynamic parameters (advanced)	Wall shear stress (WSS)	Force exerted by blood flow on vessel wall (via CFD)	Explains plaque localization and progression
	Flow patterns	Presence of recirculation zones/turbulence	Associated with lesion progression and restenosis risk
Procedural planning tools	Landing zone identification	Disease-free reference segments	Reduces geographic miss and edge dissection
	Virtual stenting simulation	Computational modeling of stent deployment	Assists in strategy selection (provisional vs. two-stent)
	CT–fluoroscopy fusion	Overlay of CCTA with angiography	Improves procedural guidance (investigational)

## Data Availability

No new data were created or analyzed in this study. Data sharing is not applicable to this article.
